# Tick’s Nest in Auditory Canal

**DOI:** 10.1093/omcr/omae160

**Published:** 2025-01-18

**Authors:** Hann-Ziong Yueh, Shih-Chun Lu, Hung-Lun Chu, Che-Hsuan Lin

**Affiliations:** Department of Otolaryngology, Taipei Medical University Hospital, Taipei Medical University, Taipei 11031, Taiwan; Department of Otolaryngology, Taipei Medical University Hospital, Taipei Medical University, Taipei 11031, Taiwan; Department of General Medicine, Taipei Medical University Hospital, Taipei 11031, Taiwan; Department of Otolaryngology, Taipei Medical University Hospital, Taipei Medical University, Taipei 11031, Taiwan; Department of Otolaryngology, School of Medicine, College of Medicine, Taipei Medical University, Taipei 11031, Taiwan

**Keywords:** ticks, otoacariasis, auditory canal

## Case report

**Figure 1:**
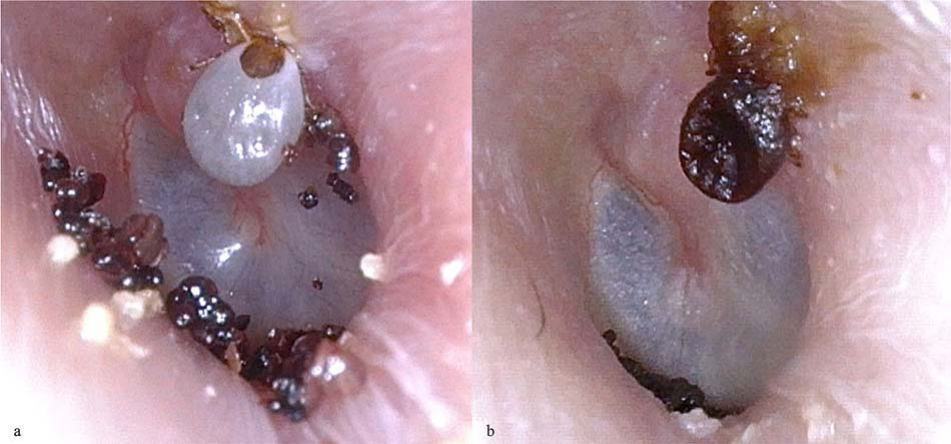
(a) A clearly visible tick attached to the upper wall of the external auditory canal, surrounded by numerous scattered eggs on the canal floor, resembling a nest within a cave; (b) post-treatment otoscopic image showing the result after using antibacterial ear drops.

A 73-year-old female, residing in the mountains and frequently sharing her bed with her cat, presented to the Department of Otolaryngology with complaints of the foreign body sensation in her left ear. She denied otalgia, tinnitus, or hearing loss. Otoscopic examination revealed a vividly visible, engorged tick firmly attached to the upper wall of the left external auditory canal. Surrounding the tick were numerous scattered eggs, creating a striking image resembling a nest within a cave (Fig. 1a). The tympanic membrane was intact. The patient was prescribed Neomycin/Polymyxin B/Lidocaine ear drops, 3 drops twice daily for 5 days, and an outpatient follow-up was arranged for the removal of the tick and eggs. A post-treatment otoscopic image (Fig. 1b) showed the condition after ear drops use, and the tick was extracted using a day hook under a microscope. The procedure was meticulously performed to avoid any damage to the surrounding tissues, ensuring the complete removal of both the tick and its eggs. Post-procedure, the patient remained asymptomatic with no signs of systemic illness, and the ear canal appeared clear and healthy. During a one-month follow-up visit, the patient reported no complications and had fully recovered.

Otoacariasis involves the attachment of ticks and mites within the ear canal. The ears are a common site for these parasites due to their accessibility and favorable conditions for tick survival [1]. The primary complications associated with intra-aural foreign bodies include canal abrasion, laceration, or bleeding. More severe conditions can also arise, such as otitis externa, tympanic membrane perforation, and suppurative otitis media, affecting the middle ear. If left untreated, these conditions can escalate, leading to chronic ear infections and potential hearing loss [2]. This case presents a vivid and complete image, showing both the engorged tick and the numerous eggs laid within the ear canal, forming what resembles a nest.
